# Organic Pollution in Surface Waters from the Fuglebekken Basin in Svalbard, Norwegian Arctic

**DOI:** 10.3390/s110908910

**Published:** 2011-09-15

**Authors:** Żaneta Polkowska, Katarzyna Cichała-Kamrowska, Marek Ruman, Krystyna Kozioł, Wiesława Ewa Krawczyk, Jacek Namieśnik

**Affiliations:** 1 Department of Analytical Chemistry, The Chemical Faculty, Gdansk University of Technology, 11/12 Narutowicza St., Gdansk 80–233, Poland; E-Mails: katarzyna.cichala@wp.pl (K.C.-K.); chemanal@pg.gda.pl (J.N.); 2 Faculty of Earth Sciences, University of Silesia, 60 Będzińska St., Sosnowiec 41–200, Poland; E-Mails: marek.ruman@us.edu.pl (M.R.); wieslawa.krawczyk@us.edu.pl (W.E.K.); 3 Department of Geography, University of Sheffield, Winter Street, Sheffield S10 2TN, UK; E-Mail: k.a.koziol@gmail.com (K.K.)

**Keywords:** polycyclic aromatic hydrocarbons, polychlorinated biphenyls, surface waters, Fuglebekken basin, Svalbard, Norwegian Arctic

## Abstract

The Fuglebekken basin is situated in the southern part of the island of Spitsbergen (Norwegian Arctic), on the Hornsund fjord (Wedel Jarlsberg Land). Surface water was collected from 24 tributaries (B1–B24) and from the main stream water in the Fuglebekken basin (25) between 10 July 2009 and 30 July 2009. The present investigation reveals the results of the analysis of these samples for their PAH and PCB content. Twelve of 16 PAHs and seven PCBs were determined in the surface waters from 24 tributaries and the main stream. Total PAH and PCB concentrations in the surface waters ranged from 4 to 600 ng/L and from 2 to 400 ng/L respectively. The highest concentrations of an individual PCB (138–308 ng/L and 123 ng/L) were found in samples from tributaries B9 and B5. The presence in the basin (thousands of kilometres distant from industrial centres) of PAHs and PCBs is testimony to the fact that these compounds are transported over vast distances with air masses and deposited in regions devoid of any human pressure.

## 1. Introduction

The contamination of the aquatic environment by stable organic compounds like polycyclic aromatic hydrocarbons (PAHs) or polychlorinated biphenyls (PCBs) is giving cause for alarm worldwide [1,2]. Because of their properties, these compounds can not only occur in water; they can be deposited in sediments or accumulate in the tissues of aquatic animals and can also be metabolized to compounds that are even more toxic and/or carcinogenic [2,3]. PAHs may turn up in the aquatic environment as a result of natural events—forest fires, volcanic eruptions, natural leakage, diagenesis of organic matter, synthesis by plants; but their presence there may also be anthropogenic: petrogenic (emergencies and leakage from the extraction and processing of crude oil and its products) and combustion (incomplete combustion at high temperatures and pyrolysis of organic matter) [4–6]. On the other hand, the presence of PCBs is solely due to human agencies. Although the production of PCBs has ceased in many countries, they may still be present in the environment. PCBs are emitted from anthropogenic sources, such as historical intentional production, utilization, disposal and accidental releases of products or materials containing these compounds. The incidental or unwanted formation of PCBs, e.g., de novo synthesis in combustion processes, thermal process of chlorine-containing materials or combustion of by-products, such as fuel combustion, waste incineration, the oxidation of iron, spills, accidents and emergencies should be also treated as a possible environmental threat [1,7–12]. The issue of the transport and fate of PAHs and PCBs substances in remote areas has received increasing attention during the past decade. Long-range atmospheric transport, deposition and air/water exchange are key processes governing the distribution of PAHs and PCBs on a global scale [2]. PAH and PCB pollutants enter the surface waters, as a result of wet and dry deposition, and via runoff from area sources (industry, households).

The Arctic, regarded in the past as a pristine area in terms of anthropogenic pollution, has nowadays become an area of great concern. Previous studies have demonstrated that this region is a significant recipient of various groups of persistent toxic substances originating from countries in both the Northern and Southern Hemispheres [2]. In the Arctic PAHs have been detected and determined in samples from the abiotic environment: snowpit (Greenland) [13,14], lake sediments (Svalbard) [2,15] and sea sediments (Barents Sea) [16]. PCBs have been determined far more frequently in samples from the Arctic, e.g., in lake sediments (Svalbard, Bjørnøya, Canadian Arctic, Russia, Greenland) [2,15,17–22]. PAHs have been determined in various tissues and organs from wildlife (Svalbard, Bjørnøya, Hornøya, Barents Sea, Franz Josef Land, Jan Mayen, Vestfjorden, Lofoten, Greenland, North Sea) [16,17,23–25].

PAHs and PCBs are highly toxic and are included in the priority substance list of the *Water Framework Directive*. Although more than half of PAH compounds are not carcinogenic, distribution of PAHs and PCBs in the environment as well as the associated potential health risks have become the focus of much attention. Their presence in Svalbard surface waters in combination with other potentially toxic compounds could have deleterious effects, which is why defining the source of origin of these contaminants in the environment is a matter of urgency.

In respect to the discussion of the PAHs one needs to emphasize that more than half of them shown are non-carcinogenic, and that the same applies to their gas phase/particulate phase ratio: naphthalene, phenanthrene, anthracene, acenaphth(yl)ene are fully in the gas phase and not associated with the particulate phase (dust); this starts with fluoranthene.

The Fuglebekken basin is situated in the southern part of the island of Spitsbergen, on the Hornsund fjord (Wedel Jarlsberg Land). It has an area of *ca.* 2–3 km^2^. It includes the steep slopes of the Ariekammen and Fugleberget, as well as a large part of the Fuglebergsletta plain together with the raised beach. The highest point of the basin lies at 568.7 m above sea level, whereas the lowest lies at sea level (the average height is 284.35 m). Gradients in the basin are very steep (400.49‰). In the upper part of the basin there are several streams, which join into one at the point where it crosses the berm—this is where the water level recorder was installed. One of the tributaries drains a small lake that formed behind the berm. Below the measurement station, the stream takes the form of an anastomosing river. The Fuglebekken main stream debouches into the Isbjörnhamna, cutting across the stony beach. In dry periods, the outflow may be completely concealed beneath boulders. The hydrologically active part of the year in the Fuglebekken lasts for 145 days. Pulina *et al.* [26] describe the hydrological season in the Fuglebekken, dividing it into three periods: snowmelt and rapid outflow (until mid-July), medium and small flows strictly due to precipitation, and the autumn-winter period of large flows due to intensive precipitation. Hydrological measurements made in the Fuglebekken during an expedition in 1979–1980 indicated a mean flow in the stream (excluding periods when no water flowed) of 0.082 m^3^·s^−1^. The outflow at this time was 822.2 mm, which was 95% of the total precipitation (864.5 mm; Pulina *et al.* [27]). The shape and location of this basin make it a very interesting object of study. The presence in this basin (thousands of kilometres distant from industrial installations) of PAHs and PCBs provide evidence for their having been transported over vast distances with air masses and their deposition in areas wholly devoid of any pressure from human agencies.

## 2. Experimental Section

### 2.1. Sampling and Site Description

**Figure 1.**
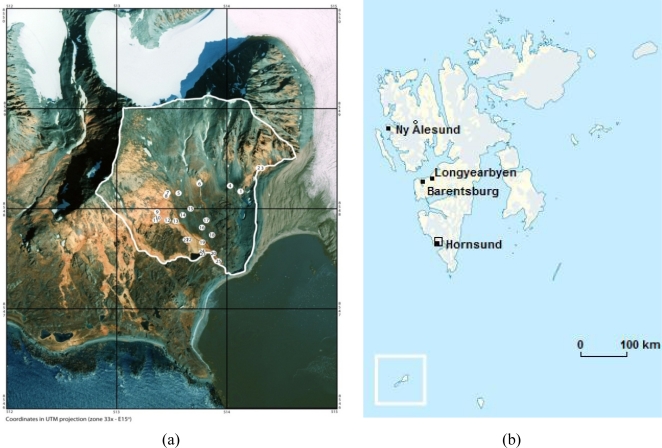
Location map for the sampling area: (**a**) surface water sampling points in the Fuglebekken basin (Kolondra L., Norway, Svalbard, Spitsbergen, Orthophotomap 1:10,000, NPI-TRomso University of Silesia); (**b**) major settlements in Svalbard and Polish Polar Station in Hornsund (labelled ‘Hornsund’) (http://en.wikipedia.org/wiki/Template:Location_map_Svalbard).

Surface water was collected from 24 tributaries (B1–B24) and from the main stream in the Fuglebekken basin (25). Location map for the sampling area (Fuglebekken basin, marked with black frame). Major settlements in Svalbard and Polish Polar Station in Hornsund (labelled ‘Hornsund’ are shown on Figure 1. The details of the sampling locations are given in Table 1.

The main stream water (Fuglebekken) was sampled between 10 July 2009 and 14 September 2009; tributaries B1–B24 were sampled on 30 July 2009. The Fuglebekken has an area of 2.02 km^2^ (real surface area 2.64 km^2^). Geologically the basin belongs to the Hecla Hoek formation, composed of metamorphic rocks; those within the basin were formed during the Proterozoic (Hjelle [28]). The basin consists entirely of rocks of the Ariekammen group of the Isbjörnhamna formation.

According to Pękala [29], every year some 340–580 g of weathered rock material comes away from every m^2^ of the surface of the nunataks north of Hornsund. There are extensive alluvial fans at the base of the slopes in the northern part of the basin. The remaining, flat part is a raised beach. In places structural soils have formed on its surface in the form of rocky rings (in the NE). The former berm, of marine pebbles, also crosses the basin. The western watershed of the Fuglebekken consists of low rocks, morphologically interpreted as roches moutonées. To the east the basin borders on the lateral moraine of the Hans Glacier, embracing the outwash plain on a rocky substrate. The total length of the watershed is 6.3 km, and its mean slope is 90.27‰.

The basin’s morphology can be characterized by means of shape indices. With a length of 2.1 km and an average width of 0.96 km, the form factor is 0.45. Table 2 lists the parameters of the Fuglebekken river basin in detail.

The area adjacent to the basin is where the meteorological station operated by the Polish Polar Station is situated. The mean annual temperature there in 1978–2006 was −4.4 °C. The maximum mean monthly temperature occurs in July (4.4 °C), the minimum in January (−11.2 °C). In 1979–2006 the mean annual precipitation was 430 mm, and the snow cover (av. depth 20 cm) lasted from an average of 241 days in the year.

### 2.2. Chemicals

All solvents used for sample processing and analyses were GC-pure quality and were purchased from the Sigma-Aldrich Company (dichloromethane-PESTANAL^®^, solvent for residue analysis; methanol-CHROMASOLV^®^, for HPLC, ≥99.9%; hexane-CHROMASOLV^®^, for HPLC, ≥97.0% (GC)). A mixture of 16 PAHs and two deuterated internal standards (naphthalene-d_8_, benzo(a)anthracene-d_12_) in dichloromethane at a concentration of 2,000 μg/mL for each were from Restek Corporation (USA) and Supelco (USA) respectively. A working stock solution was prepared from seven selected PCB standards (IUPAC Nos. 28, 52, 101, 118, 153, 138 and 180). Standards were also purchased from Restek Corporation (USA) as 10 μg/mL solutions in isooctane. Certified standards of ^13^C-labelled PCB 28 and PCB 180 (40 μg/mL in nonane) were obtained from Cambridge Isotope Laboratories (USA) [30,31].

### 2.3. Analytical Procedure

**Figure 2.**
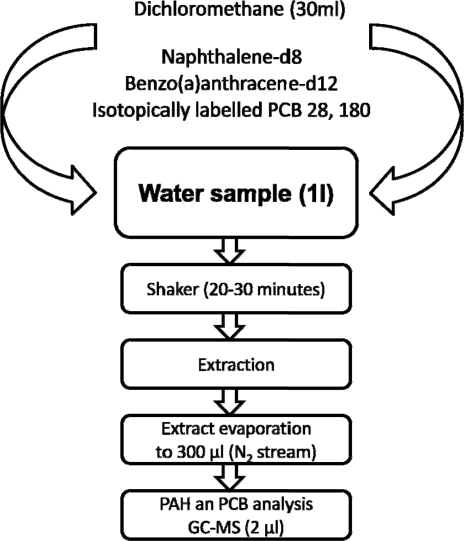
Flow chart of the procedure for preparing sea water samples for determining their PAH and PCB contents.

A 1 L sample of water was extracted with 30 mL dichloromethane, two deuterated internal standards (naphthalene-d_8_ (*m/z* 136) and benzo(a)anthracene-d12 (*m/z* 240)), and two isotopically labelled internal standards of PCB 28 (*m/z* 270, 268) and PCB 180 (*m/z* 408, 406) and shaken for 20–30 min. After liquid-liquid extraction (LLE), the extract was evaporated to a volume of 300 μL under a gentle stream of nitrogen (Figure 2) [30–34].

The final extracts were analysed using an Agilent Technologies 7890A gas chromatograph with an Agilent Technologies 5975C mass spectrometric detector and split/splitless injector (7683B). A ZB-5MS capillary column (5% phenyl + 95% dimethylpolysiloxane, 30 m × 0.25 mm × 0.25 μm) was used. The temperature programme was the following: initial temperature 40 °C, 40 °C to 120 °C at 40 °C min^−1^, then 120 °C up to 280 °C at 5 °C min^−1^ where it was held for 17 min (PAHs) and for 5 min (PCBs). The carrier gas was helium with inlet pressure 70 kPa. The injection volume selected for all analyses was 2 μL. The mass spectrometer was operated in the selected ion monitoring (SIM) mode. The following ions were monitored: (m/z) PAH: 128, 127, 152, 151, 154,153, 166, 165, 178, 176, 203, 202, 228, 226, 252, 250, 277, 276, 279 and 278, and PCB: 258, 256, 292, 290, 328, 326, 362, 360, 396, and 394 [35,36]. Before sample analysis, the relevant standards were analysed to check column performance, peak height and resolution, and the limits of detection and quantification. A solvent blank, a standard mixture and a procedural blank were run in each sequence of samples to check for contamination, peak identification and quantification. Compounds were identified mainly by their retention times. Measuring range, detection and quantification limits are presented in Table 3.

## 3. Results and Discussion

### 3.1. Levels of PAHs in Surface Waters and an Indication of Their Origin

The concentrations of 12 of 16 PAHs in surface water from 24 tributaries and stream water (Fuglebekken) are shown in Table 4. These compounds were determined at 17 of the 25 surface water sampling points, at concentrations ranging from 4 to 600 ng/L. The highest concentration of an individual PAH (naphthalene, 557 ng/L) was found in a sample from tributary B5.

Points B5, B6 and B7, where decidedly higher PAH levels were detected and determined in the samples, lie at the foot of a mountain down which meltwater from snow and ice flows. These waters flush out pollutants from the air and also from the mountainsides, where on days without precipitation contaminants are deposited that arrived there with dusts. These contaminants are then carried by the tributaries to the Fuglebekken main stream. In those parts of the basin farthest away from the mountain range PAH levels were much lower as a result of their having been diluted with cleaner stream water. Moreover, points B5–B7 are situated in erosion hollows (gullies), where snow accumulates and lies.

The spatial distribution of PAH concentrations showed considerable variation within the study area, and there were clear differences in residue levels between sampling points. Samples from points B1–B4, at the edge of the basin, contained very different levels of naphthalene, where these values were well below 1 ng/L. In samples taken elsewhere in the basin naphthalene levels were from a few to several hundred ng/L, whereas acenaphthylene, anthracene and fluorene were found more frequently at those points.

The occurrence of high PAH levels in these areas might be due to their proximity to a coal mine. Mining and excavation activities took place at this site for a long time; the excavated products were transported by train from the mine site to a dock, and some of the coal was used to run a power station. Although the mining operation was closed in 1962, railways and coal heaps remain in the area and could be contributing to the elevated levels of PAHs in the Fuglebekken.

To date, no analysis of surface water samples from Svalbard for the presence of PAHs has been carried out. Even so, the compounds have been detected in bottom sediments in Ny-Ålesund, Wijdefjorden and Adventfjorden [2,16,18], and also in ambient particulate matter [15], surface soil [17], moss [17] and reindeer dung [17]. PAHs have also been detected and determined in bivalves samples from Svalbard [16] (Table 5). The PAH most commonly determined in bottom sediments on Svalbard was naphthalene [2], just as in the water samples from the Fuglebekken.

The highest PAH level measured in the 1995 survey (191 ng/g dry wt) was found in sediment from Tenndammen, which is affected by coal combustion and the diesel power plants in Longyearbyen (Svalbard). Together with the recent upsurge in research activities and the establishment of research stations, the number of cruises and ships to Ny-Ålesund increased remarkably during 1996–2005. Total diesel fuel and petrol consumption by power stations and vehicles also increased slightly from 2001 to 2005. Consumption of diesel in the power stations in 2001 and 2005 was 1,009 and 1,130 m^3^ respectively, and that of vehicle fuels was 34 m^3^ in 2001 and 37 m^3^ in 2005 [2].

Trends in the intensity of PAH deposition, evaluated from cores taken from the Arctic ice cap, have shown a dramatic increase in concentrations over the last 100 years that correlate well with the historical record of world petroleum production. An increase in the concentration of PAHs relative to those of fatty acids of terrestrial higher plant origin (C_20_–C_32_) demonstrated that contributions of anthropogenic PAHs have increased significantly since the 1930s. Similarly, other researchers have concluded from analyses of surface snow samples, also taken from the Arctic ice cap, that current PAH contamination is essentially due to fossil fuel combustion with some input from biomass burning [23].

PAHs are present in the Arctic environment from both anthropogenic and natural sources (petrogenic/biogenic). The literature makes reference to various methods of identifying sources of PAH emissions that are based on concentration ratios of particular compounds, for example, the ratio of a substituted compound to an unsubstituted one [12,35,37]. Petrogenic PAHs are formed slowly, over a long time, at moderate temperatures (100–300 °C), and are associated with fossil fuels (petroleum and coal). Combustion PAHs are formed during rapid, high-temperature combustion (>700 °C) of motor (automobile), bunker (shipping), and power plant (coals and petroleum) fuels. Combustion PAHs are also formed at the intermediate temperatures (400−600 °C) reached in the processing of coals into coal tars and coal tar products (e.g., creosote, or the coal tar pitch used in aluminium smelters). Thus, the characteristic PAH compositional profile of these different sources can be used to help distinguish them [36].

To further assess the potential sources of PAHs to the surface waters from Svalbard, PAH indicator ratios, commonly used to trace the origin of PAHs, were considered [35,37]. Several PAH indicator ratios in surface water sediments were compared to those in some typical sources reported in the literature (Table 6).

Phenanthrene/anthracene, the most commonly used ratio, ranged from 0.001 to 0.908 (mean 0.7) in surface water, which may suggest that the majority of PAHs determined in samples of water taken in various places in the Fuglebekken was derived from combustion sources. This is the same ratio as for lake sediments in Svalbard, which may suggest that the majority of PAHs in the lakes were derived from the same sources. On the other hand, although the number of samples was limited, the PAH ratios of coastal marine sediments (Svalbard) suggest petrogenic origins [2].

Relatively high fluoranthene/(fluoranthene + pyrene) ratios are also typical of aerosols associated with combustion PAHs [2,23]. The ratio characteristic of samples of water from the Fuglebekken indicate that PAHs probably originate both from local sources due to the burning of fossil fuels and high temperature biomass combustion, as well as from aerosol inputs through long-range atmospheric transport.

Table 7 sets out information that may be of help in interpreting the measurement data (Table 6). It shows that most of the contamination comes from the combustion source: for the samples from the tributaries (B1–B24), 99% is due to combustion and 1% to petrogenic influence. The situation regarding stream water is very similar. More than 70% of the samples are of combustion origin; the remainder is the result of petrogenic influence.

### 3.2. Levels of PCBs in Surface Waters and an Indication of Their Origin

The concentrations of 7 PCBs in samples of surface water from 24 tributaries and stream water (Fuglebekken) are shown in Table 8. These compounds were determined in samples taken from 20 of the 25 sampling points. Total PCB concentrations in surface water ranged from 2 to 400 ng/L. The highest concentrations of an individual PCB 138 (308 ng/L and 123 ng/L) were found in samples from tributaries B9 and B5. At these points the levels of PCB 101 and 118 were also high.

As far as PCBs are concerned, there are no data in the literature on markers that could indicate the sources of their emission. This is primarily because PCBs are synthesized chemically, so that in the environment they occur mainly as mixtures containing various amounts of their congeners. Hence, it is not possible to determine their points of origin on the basis of molecular ratios. Nevertheless, if we have data on PAHs at our disposal, by drawing comparisons and seeking mutual correlations we can “define” the probable source of emission of PCB compounds [36].

### 3.3. Loads of Organic Compounds Transported by the Fuglebekken Stream

The levels of organic pollutants in the Fuglebekken varied: in proportion to the volume of water carrier by each tributary of the basin’s main stream, these levels reflect the overall load of PAHs and PCBs that it carries into the Hornsund fjord. Table 10 lists the loads of PCBs and PAHs transported in the Fuglebekken basin.

The total load of PCBs present at the outlet of the Fuglebekken ranged from 0.06 mg per 24 h in the case of PCB-52 to 0.60 mg per 24 h in the case of PCB-118. The largest PCB loads in the main stream were recorded for the pentachlorobiphenyls PCB-101 and PCB-118. These two compounds made up to 6.4% of the total PCB load in the Fugle stream, while the measured congeners encompassed 14% of the total PCB load. The overall PCB load carried in 24 h is 17.9 mg, which is equivalent to the transport of 745 μg PCBs across the stream’s cross-section in 1 h. The load of PAHs in the Fuglebekken is half as great (9.90 mg per 24 h, 420 μg per h). The significant compounds among the PAHs were naphthalene, acenaphthylene and pyrene (0.89, 0.44 and 0.31 mg per 24 h respectively).

## 4. Conclusions

PAHs and PSBs were detected and determined in the waters of the Fuglebekken basin in Svalbard, Norwegian Arctic. In the case of PAHs it is easy to pinpoint the source on the basis of the concentration ratios of the relevant compounds. The presence of PAHs in Svalbard may be due to long-distance transport, and also to the combustion of fuel for transportation, heating and electric power production purposes at the Polish Polar Station. In the case of long-distance transport, taiga fires are also a probable source, as PAHs are also produced during the combustion of biomass. It takes just a few days for pollutants from Europe to reach the Arctic, and within a few weeks the toxins may have entered the food chain. Contaminants are transported much more slowly in sea water, carried as they are by sea currents: it is thought that it takes 30−40 years for contaminants to reach the Arctic by this means. Because of their lipophilic properties, PAHs are more commonly found in soils and sediments [2,18] than in waters. It is therefore likely that the PAH levels in the soils of the Fuglebekken basin are higher than those measured in the stream water. PAH levels in waters may also be affected by the rock load they are carrying and by dry atmospheric deposition. These compounds get into the basin mostly with precipitation, usually snow, which often lies for very long periods among the mountain peaks. Here, PAHs can be deposited together with dust that has been carried even for thousands of kilometres. Contaminants are transported to and within the Arctic along various routes. A major pathway is the atmospheric transport of contaminants from mid-latitudes to the Arctic region. In winter, areas of high pressure are present over the continents and of low pressure over the northern Pacific and Atlantic Oceans; the intense Siberian high-pressure cell tends to force air on its western side northwards into the Arctic. Consequently, airborne pollutants are transported from sources in Eurasia into the Arctic. In summer, the Eurasian flow is reversed, resulting in a weak north to south transport [65].

In contrast, the source of PCBs cannot be determined with any certainty. Given the current state of knowledge, however, it is possible, if we have levels of both PAHs and PCBs, to draw indirect inferences regarding the source of these latter compounds in the environment; this is also possible by comparing or seeking suitable correlations between PAH and PCB levels, and on the basis of information obtained from hydrological, meteorological and historical sources. PCBs may be present in Svalbard surface waters as a result of the long-range transport of air masses: the well known Arctic haze—a manifestation of the accumulation of atmospheric pollutants in the Arctic—is due to the minimal exchange of air with non-polar areas in winter. In the past these compounds were used in electrical equipment such as transformers, condensers and switches. The fact that such equipment was used at the Polish Polar Station could have led to the contamination of the running waters in the vicinity, especially if the reservoir from which these substances are released is the soil, however the results of recent studies indicate that levels of PCBs in the tissues of sea birds in the Arctic are falling, a trend that is expected to continue. Even so, PCBs, whose environmental content is decreasing only very slowly, continue to be the most serious pollutants of the Arctic.

## Figures and Tables

**Table 1.** The details of the sampling locations.

***Site***	***Position***	
	***latitude***	***longitude***

***Tributary***		
***B 1***	77.011922	15.562937
***B 2***	77.013944	15.570209
***B 3***	77.013944	15.570149
***B 4***	77.012417	15.559140
***B 5***	77.011758	15.540471
***B 6***	77.012645	15.548105
***B 7***	77.011767	15.536465
***B 8***	77.011566	15.536151
***B 9***	77.010115	15.532617
***B 10***	77.009535	15.532708
***B 11***	77.009388	15.532094
***B 12***	77.009351	15.536401
***B 13***	77.009263	15.539309
***B 14***	77.009824	15.541948
***B 15***	77.010412	15.544770
***B 16***	77.008702	15.548806
***B 17***	77.009332	15.550478
***B 18***	77.008005	15.552410
***B 19***	77.007351	15.548917
***B 20***	77.006343	15.552868
***B 21***	77.007593	15.543833
***B 22***	77.007592	15.543894
***B 23***	77.006420	15.548989
***B 24***	77.006501	15.548812
***Stream water***		
***B 25***	77.005856	15.553388

**Table 2.** The parameters of the Fuglebekken basin.

**Parameter**	**Value**
Maximum height above sea level	568.7 m
Minimum height above sea level	0.0 m
Relative height above sea level	568.7 m
Mean height of basin	284.35 m
Slope of basin	400.49‰
Slope of watershed	90.27‰
Area of basin	2.02 km^2^
Real area of basin	2.64 km^2^
Length of basin	2.1
Length of watershed	6.3 km
Mean width of basin	0.96
Form factor	0.45
Compactness index	1.24
Circularity ratio	0.64
Elongation ratio	0.76
Lemniscate index	0.5

**Table 3.** Measurement range, detection and quantification limits for the final determination of PAHs and PCBs.

***Analytes***		***Measuring range [ng/L]***	***LOD [ng/L]***	***LOQ [ng/L]***
*PCBs*		0.02–560	0.025	0.075
	Naphthalene	0.034–560	0.034	1.02
	Acenaphthylene	0.0041–560	0.0041	0.0123
	Acenaphthene	0.0041–560	0.0041	0.0123
	Fluorene	0.0018–560	0.0018	0.0054
	Phenanthrene	0.0025–560	0.0025	0.0075
	Anthracene	0.0078–560	0.0078	0.0234
	Fluoranthene	0.014–560	0.014	0.042
*PAHs*	Pyrene	0.028–560	0.028	0.084
	Chrysene	0.0022–560	0.0022	0.0066
	Benzo(b)fluoranthene	0.014–560	0.014	0.042
	Benzo(k)fluoranthene	0.0023–560	0.0023	0.0069
	Benzo(a)pyrene	0.0055–560	0.0055	0.0165
	Benzo(a)anthracene	0.0017–560	0.0017	0.0051
	Indeno(1,2,3-cd)pyrene	0.43–560	0.43	1.29
	Dibenz(a,h)anthracene	0.014–560	0.014	0.042

**Table 4.** Concentrations (ng/L) of PAHs in samples of water from Hornsund taken near the Polish Polar Station, Svalbard.

***PAH compound***		***Nap***	***Acy***	***Ace***	***Fl***	***Phe***	***Ant***	***Flr***	***Pyr***	***Chy***	***BbF***	***BkF***	***BaP***	***Total PAHs***

***LOCATION***														
***Tributary***														

*B1*		0.12	3.4	0.26	<0.0018	<0.0025	11	<0.014	<0.028	<0.0022	<0.014	<0.0023	<0.0055	15
*B2*		0.034	0.27	0.061	0.24	<0.0025	4.4	2.0	13	0.19	<0.014	<0.0023	0.2	20
*B3*		0.066	2.5	1.3	2.3	<0.0025	25	<0.014	<0.028	<0.0022	<0.014	<0.0023	<0.0055	31
*B4*		<0.034	1.7	1.9	2.0	<0.0025	53	2.8	4.2	<0.0022	<0.014	<0.0023	<0.0055	65
*B5*		557	1.1	0.98	0.93	0.23	31	1.8	<0.028	1.7	<0.014	9.0	<0.0055	603
*B6*		310	1.7	1.5	<0.0018	<0.0025	26	<0.014	<0.028	<0.0022	<0.014	<0.0023	<0.0055	339
*B7*		16	<0.0041	<0.0041	<0.0018	<0.0025	<0.0078	<0.014	<0.028	<0.0022	<0.014	<0.0023	<0.0055	16
*B8*	*N = 1*	3.0	<0.0041	0.98	<0.0018	<0.0025	<0.0078	<0.014	<0.028	<0.0022	<0.014	<0.0023	<0.0055	4.0
*B9*		0.51	<0.0041	1	1.5	<0.0025	16	2.5	6.1	<0.0022	11	11	<0.0055	50
*B10*		11	<0.0041	<0.0041	<0.0018	<0.0025	<0.0078	<0.014	<0.028	<0.0022	<0.014	<0.0023	<0.0055	11
*B15*		42	<0.0041	1.1	1.4	<0.0025	14	1.5	<0.028	<0.0022	<0.014	<0.0023	<0.0055	60
*B17*		11	<0.0041	3.3	1	<0.0025	67	<0.014	<0.028	<0.0022	<0.014	<0.0023	<0.0055	83
*B18*		110	<0.0041	<0.0041	<0.0018	<0.0025	<0.0078	<0.014	<0.028	<0.0022	<0.014	<0.0023	<0.0055	110
*B19*		8.8	<0.0041	<0.0041	<0.0018	<0.0025	<0.0078	<0.014	<0.028	<0.0022	<0.014	<0.0023	<0.0055	8.8
*B20*		<0.034	<0.0041	0.94	<0.0018	<0.0025	7.5	<0.014	<0.028	<0.0022	<0.014	<0.0023	<0.0055	8.4
*B22*		13	<0.0041	0.55	<0.0018	<0.0025	4.5	1.4	<0.028	<0.0022	<0.014	<0.0023	<0.0055	20

***Stream water***														
*Fuglebekken*	*N = 70*	0.74	1.5	0.055	0.0094	0.074	0.23	0.099	0.52	0.019	0.04	0.014	0.025	3.3

Naphthalene (Nap), Acenaphthylene (Acy), Acenaphthene (Ace), Fluorene (Fl), Phenanthrene (Phe), Anthracene (Ant), Fluoranthene (Flr), Pyrene (Pyr), Chrysene (Chy), Benzo(b)fluoranthene (BbF), Benzo(k) fluoranthene (BkF), Benzo(a)pyrene (BaP).

**Table 5.** Concentrations of PAHs in abiotic and biotic samples from Svalbard.

	***Location***	***Object***		***Concentration***	***References***

				[ng/g] *	
***Abiotic environment***	*Svalbard*				
	*Ny-Ålesund*	***Sediments***		Σ_15_PAHs 27–711 (lakes) (dw)	[2]
				Σ_15_PAHs 27–34 (coastal area) (dw)	
		***Ambient particulate matter***		ΣPAHs 0.6–2.0 ng/m^3^	[15]
		***Surface soil***		Σ_16_PAHs 37–324 (dw)	[17]
		***Moss***		Σ_16_PAHs 158–244 (dw)	
		***Reindeer dung***		Σ_16_PAHs 49–340 (dw)	
	*Wijdefjorden*	***Sediments***		Σ_16_PAHs 36 (fjords, tidal plains) (dw)	[18]
				Σ_16_PAHs 429 (lakes) (dw)	
	*Adventfjorden*			Total PAH 0.94 (ww)	[16]

	***Location***	***Species***	***Tissue***	***Concentration***	

***Biotic environment***				[ng/g] *	
	*Svalbard*				
	*Adventfjorden*	***Bivalves***	*C*	Total PAH 0.20 (lw)	[16]

* concentrations reported as means or ranges of means of dry weight (dw), wet weight (ww) or lipid weight (lw); C—clam.

**Table 6.** PAH indicator ratios for surface water samples from Svalbard and the potential sources of the emission of these compounds.

**PAH ratio**	**Nap/Phe**	**Phe/Ant**	**Ant/(Ant+Phe)**	**BaA/Chy**	**BaA/(BaA+Chy)**	**Flr/Pyr**	**Flr/(Flr+Pyr)**	LMW/HMW *	**Fl/(Fl+Pyr)**	**BaP/(BaP+Chy)**	**BbF/BkF**
***LOCATION***											
***Tributaries***											
*B1–B24*	2,476	0.000668	0.999 (0.993–1)	–	–	0.412 (0.156–0.669)	0.638 (0.135–1)	56.4 (1.26–102)	0.648 (0.0181–1)	0.509	1.015
***Main stream water***											
*Fuglebekken*	96 (0.409–595)	0.771 (0.0207–0.908)	0.851 (0.524–1)	0.846 (0.0224–1.20)	0.547 (0.0219–0.546)	0.628 (0.0830–2.04)	0.526 (0.0766–0.671)	14.2 (0.0261–241)	0.755 (0.00431–1)	0.773 (0.235–1)	3.97 (0.140–8.19)

***SOURCE***											
*Combustion*	>1^[6]^	<10^[38,39]^	>0.1^[42]^	>1^[43]^	>0.2^[45]^	>1^[38]^	0.4–0.5^[45]^	<1^[38,47]^			
				>0.9^[43]^			>0.5^[45]^				
*Petrogenic*	<1^[6]^	>10^[38]^	<0.1^[42]^	<1^[43]^	<0.2^[45]^	<1^[38]^	0.22^[45]^	>1^[38,47]^			
		>15^[39]^		<0.4^[43]^							
		>25^[40]^									
Petrol emissions			0.47–0.59^[3,44]^			0.43^[41]^		<0.5^[44,48–51]^	0.73^[52,53]^	1.07–1.45^[3]^	
*Diesel*									>0.5^[44,48–51]^	0.5^[52,53]^	>0.5^[54,55]^
Coal emissions			1.05–1.17^[3,44]^			0.58^[41]^				3.53–3.87^[3]^	
*Fuel oil*		50^[41]^				0.9^[46]^					

* LMW/HMW—Low Molecular Weight/High Molecular Weight.

**Table 7.** Interpretation of measurement data regarding the main sources of PAH emission found in water samples from Svalbard.

**PAH ratio**		**Fuglebekken [%]**	**B1–B24 [%]**	**Source**
Nap/Phe	>1	91	100	**combustion**
	<1	9	-	petrogenic
Phe/Ant	<10	100	100	**combustion**
Ant/(Ant+Phe)	>0.1	100	100	**combustion**
BaA/Chy	>1	71	-	**combustion**
	<0.4	29	-	petrogenic
BaA/(BaA+Chy)	>0.2	90	-	**combustion**
	<0.2	10	-	petrogenic
Flr/Pyr	>1	15	-	combustion
	<1	85	100	petrogenic
Flr/(Flr+Pyr)	>0.4	58	100	**combustion**
	<0.4	42	-	petrogenic
LMW/HMW	<1	64	100	**combustion**
	>1	36	-	petrogenic
Fl/(Fl+Pyr)	<0.5	27	43	gasoline
	>0.5	73	57	**diesel**
BaP/(BaP+Chy)	0.73	71	-	gasoline
	0.5	29	100	**diesel**
BbF/BkF	1.07–1.45	25	-	gasoline
	>0.5	75	100	***diesel***

**Table 8.** Concentrations (ng/L) PCBs found in samples of water taken from catchment area at Hornsund, near the Polish Polar Station, Svalbard.

	
	**PCB compound**								

**LOCATION**		**PCB 28**	**PCB 52**	**PCB 101**	**PCB 118**	**PCB 138**	**PCB 153**	**PCB 180**	**Total PCBs**
***Tributary***									

*B2*		8.5	6.2	5.1	1.8	8.5	8.0	10	48
*B3*		<0.025	<0.025	6.0	3.8	<0.025	<0.025	<0.025	9.8
*B5*		<0.025	2.0	10	18	123	<0.025	<0.025	153
*B7*	*N = 1*	<0.025	3.9	3.6	3.5	<0.025	<0.025	<0.025	11
*B8*		<0.025	<0.025	24	48	71	<0.025	<0.025	143
*B9*		<0.025	<0.025	26	72	308	<0.025	<0.025	406
*B10*		<0.025	4.2	<0.025	<0.025	<0.025	<0.025	<0.025	4.2
*B11*		<0.025	5.2	<0.025	<0.025	<0.025	<0.025	<0.025	5.2
*B12*		<0.025	2.3	<0.025	<0.025	<0.025	<0.025	<0.025	2.3
*B14*		<0.025	4.1	<0.025	<0.025	<0.025	<0.025	<0.025	4.1
*B15*		<0.025	1.9	4.6	7.3	<0.025	<0.025	<0.025	14
*B16*		<0.025	3.9	<0.025	<0.025	<0.025	<0.025	<0.025	3.9
*B17*		14	5.5	2.6	3.5	<0.025	<0.025	<0.025	26
*B18*		<0.025	5.33	2.2	3.4	<0.025	<0.025	<0.025	11
*B19*		<0.025	6.4	5.6	<0.025	38	<0.025	<0.025	50
*B20*		<0.025	29	<0.025	<0.025	<0.025	<0.025	<0.025	29
*B21*		<0.025	5.2	<0.025	<0.025	<0.025	<0.025	<0.025	5.2
*B22*		<0.025	26	<0.025	<0.025	8.7	<0.025	<0.025	35
*B24*		<0.025	10	2.5	<0.025	<0.025	<0.025	<0.025	13

***Stream water***									

*Fuglebekken*	*N = 70*	0.51	0.14	0.94	0.96	0.39	0.64	0.71	4.3

There are no literature data on the determination of PCBs in surface waters on Svalbard. Nonetheless, analyses of PCBs in other compartments of the environment have been done far more often. PCBs have been found in bottom sediments (Ny-Ålesund, Wijdefjorden) [2,18], and also in the biotic environment: common eider [56], kittiwake [56], glaucous gull [56–58], black guillemot [25,59], eider [59], harbour seals [60], milk [60], pups [60], bivalves [16], Brünnich’s guillemot [59], ringed seals [61], bearded seals [61] and polar bears [61–64] (Table 9).

**Table 9.** PCB concentrations in the abiotic and biotic environment in Svalbard.

	**Location**	**Object**		Concentration [ng/g] *	**Refs.**
***Abiotic environment***	*Svalbard*				
	*Ny-Ålesund*	*Sediments*		Σ_15_PCBs 0.18–13 (lakes) (dw)	[2]
				Σ_15_PCBs 0.33–0.42 (coastal area) (dw)	
	*Wijdefjorden*			Σ_7_PCB <0.9–5.5 (fjords, tidal plains) (dw)	[18]
				Σ_7_PCB <0.1–14.6 (lakes) (dw)	

***Biotic environment***	*Svalbard*				
	*Ny-Ålesund*	*Common eider, Kittiwake, Glaucous gull*	*L,B,F*	Σ_19_PCB 0.8–24 976 (ww)	[56]
	*Barentsburg*	*Glaucous gull*	*L*	Σ_32_PCB 386–2 347 (ww)	[57]
	*Longyearbyen*	*Glaucous gull, Black guillemot, Eider*	*L*	Total PCB 40–77 750 (ww)	[59]
	*Nordaustlandet*	*Glaucous gull, Black guillemot, Eider*	*L*	Total PCB 10–21 130 (ww)	
	*Prins Karls Forland*	*Harbour seals (males, females), Milk, Pups*	*Bl*	ΣPCBs 271.6–2201.1 (lw)	[60]

	*Storfjorden*	*Brünnich’s guillemot*	*L*	Total PCB 40–180 (ww)	[59]
	*Kongsfjorden*	*Ringed and Bearded seals (males, females)*	*Bd*	ΣPCBs 159/1–624/8 (lw)	[61]
	*Southern, south-eastern, northern parts*	*Polar bears*	*S,Bc*	ΣPCBs 2220–80 300 (lw)	[62]
	*Southern, south-eastern part*	*Polar bears*	*Bp*	ΣPCBs 21–228 (ww)	[63]
	*Eastern part*	*Polar bears*	*Bp*	ΣPCBs 214,4 (ww)	[64]
	*Southern part*	*Glaucous gull, Guillemots*	*L,B,K,M, eggs*	Σ_21_PCB 118–32 300 (ww)	[58]
	*Western part*		*Bl*	Σ_102_PCB 1130–5 250 (lw)	[25]

* concentrations reported as means or ranges of means of dry weight (dw), wet weight (ww) or lipid weight (lw); L-Liver, B-Brain, F-Fat, M-Muscle, K-Kidney, Bl-Blubber, C-Clam, S-Subcutaneous, Bc-Blood cells, Bp-Blood plasma, Bd-Blood.

**Table 10.** Calculated PCB and PAH loads transported in the Fuglebekken basin.

	
	**Load**		

**Analytes**	**ng per second**	**μg per hour**	**mg per day**
Nap	5.1	18	0.44
Acy	10	37	0.89
Ace	0.38	1.4	0.033
Fl	0.065	0.23	0.0056
Phe	0.51	1.8	0.044
Ant	1.6	5.6	0.14
Flr	0.68	2.5	0.059
Pyr	3.6	13	0.31
Chy	0.13	0.47	0.011
BbF	0.28	0.99	0.024
BkF	0.099	0.35	0.0085
BaP	0.17	0.62	0.015

**Total PAHs**	115	412	9.9

PCB 28	3.5	12	0.30
PCB 52	0.69	2.5	0.060
PCB 101	6.2	22	0.54
PCB 118	6.9	25	0.60
PCB 138	2.8	9.9	0.24
PCB 153	4.1	15	0.36
PCB 180	4.8	17	0.42

**Total PCBs**	207	745	18
